# Social information facilitates learning about novel food sources in adult flower-visiting bats

**DOI:** 10.1007/s10071-023-01807-9

**Published:** 2023-07-08

**Authors:** Andreas Rose, Marco Tschapka, Mirjam Knörnschild

**Affiliations:** 1 Institute of Evolutionary Ecology and Conservation Genomics, University of Ulm, Albert-Einstein-Allee 11, 89069 Ulm, Germany; 2 Smithsonian Tropical Research Institute, Building 401 Tupper, Luis Clement Avenue, Balboa Ancon, Panama, Republic of Panama; 3 Museum für Naturkunde—Leibniz Institute for Evolution and Biodiversity Science, Invalidenstraße 43, 10115 Berlin, Germany; 4 Institute for Biology, Humboldt-Universität Zu Berlin, Invalidenstr. 42, 10115 Berlin, Germany

**Keywords:** *Glossophaga soricina*, Social transmission, Anthropogenic change, Dietary repertoire, Demonstrator-observer dyad

## Abstract

Incorporating novel food sources into their diet is crucial for animals in changing environments. Although the utilization of novel food sources can be learned individually, learning socially from experienced conspecifics may facilitate this task and enable a transmission of foraging-related innovations across a population. In anthropogenically modified habitats, bats (Mammalia: Chiroptera) frequently adapt their feeding strategy to novel food sources, and corresponding social learning processes have been experimentally demonstrated in frugivorous and animalivorous species. However, comparable experiments are lacking for nectarivorous flower-visiting bats, even though their utilization of novel food sources in anthropogenically altered habitats is often observed and even discussed as the reason why bats are able to live in some areas. In the present study, we investigated whether adult flower-visiting bats may benefit from social information when learning about a novel food source. We conducted a demonstrator–observer dyad with wild Pallas’ long-tongued bats (*Glossophaga soricina*; Phyllostomidae: Glossophaginae) and hypothesized that naïve individuals would learn to exploit a novel food source faster when accompanied by an experienced demonstrator bat. Our results support this hypothesis and demonstrate flower-visiting bats to be capable of using social information to expand their dietary repertoire.

**Supplementary Information:**

The online version contains supplementary material available at 10.1007/s10071-023-01807-9.

## Introduction

Incorporating novel food sources into the dietary repertoire is crucial for animals in order to acquire resources in changing environments. This can be easily observed in anthropogenically altered habitats, where animals expand their niche by exploiting food sources related to humans, including crops, livestock or ornamental plants (Giménez-Anaya et al. 2008; Amit et al. 2013; Kruszynski et al. 2016), by feeding on invasive species (Kottsieper et al. 2019) or even by using intentionally provided feeding stations (Tryjanowski et al. 2015).

While the use of novel food sources can be learned individually, for example by trial-and-error learning, social learning by “observation of, or interaction with, another individual or its products” can facilitate this task (Hoppitt and Laland 2013, p. 4). The mere presence of a conspecific may reduce neophobia or promote explorative behavior and thus facilitate interactions with a novel food source (social facilitation, Zajonc 1965), or learning individuals may benefit from information transfer by participating in the knowledge of a more experienced conspecific (social transmission, Hoppitt and Laland 2013). Regardless of the various mediating mechanisms (Galef and Giraldeau 2001; Galef and Laland 2005), social transmission may spread foraging-related innovations across a population and form local traditions (van Schaik 2010; Laland et al. 2011; Aplin et al. 2013, 2015).

In anthropogenically modified habitats, bats (Mammalia: Chiroptera) can be frequently observed to expand their dietary repertoire with novel food sources. For instance, insectivorous bats may shift their feeding habits in response to introduced prey (Levin et al. 2006), sanguivorous bats may parasitize on pets (Rosa et al. 2013) or livestock (Bobrowiec et al. 2015), while frugivorous and nectarivorous bats may feed on introduced crops (Parry-Jones and Augee 1991; Alpízar et al. 2020) and ornamental plants (Kruszynski et al. 2016; da Silva et al. 2017; Pellón et al. 2021), or may even exploit artificial sugar water resources like hummingbird feeders (Buecher and Sidner 2013; Maguina and Muchhala 2017).

Although bats can learn to utilize novel food sources individually, their gregariousness and longevity may facilitate the use of social learning strategies (Wright 2016). In fact, the use of social information has been documented to influence various foraging decisions in bats (reviewed in Wilkinson and Boughman 1999; Wright 2016; Prat and Yovel 2020) and the possibility of a socially facilitated incorporation of novel food sources into a bat’s dietary repertoire has been experimentally demonstrated in frugivorous and animalivorous species (e.g., Ratcliffe and ter Hofstede 2005; Page and Ryan 2006; Wright et al. 2011; Clarin et al. 2014; O’Mara et al. 2014).

However, comparable experiments are lacking for neotropical flower-visiting bats (Phyllostomidae: Glossophaginae), even though their utilization of novel food sources in anthropogenically modified habitats is often observed and even discussed as the reason why bats are able to live in some areas (Buecher and Sidner 2013; Pellón et al. 2021). Flower-visiting bats habitually feed on nectar and pollen from co-evolved bat-pollinated flowers that often share characteristics described as the chiropterophilous syndrome, including a specific unpleasant scent, good accessibility during hovering flight and the production of large amounts of relatively dilute nectar (von Helversen 1995; Tschapka and Dressler 2002). Food sources such as hummingbird feeders or introduced plants often lack these co-evolved chiropterophilous characteristics, raising the question how individual bats initially learn to exploit them.

Pallas’ long-tongued bats (*Glossophaga soricina*, Glossophaginae) are medium-sized flower-visiting bats with a large geographical distribution from Mexico to Argentina, inhabiting various habitats ranging from montane cloud-forests over lowland rainforests to deciduous dry forests and savannahs (Alvarez et al. 1991; da Rocha et al. 2018). They can be found in primary forests as well as in heavily influenced anthropogenic areas such as banana monocultures and even within cities, where they are regularly observed to drain hummingbird feeders overnight (Kruszynski et al. 2016; Alpízar et al. 2020; Pellón et al. 2021; personal observation). Although there is experimental evidence that *G. soricina* readily use social information when searching for new locations of an already known food source (Rose et al. 2016), a social transmission of dietary preferences or information about novel food sources has not been demonstrated yet (Rose et al. 2019).

In the present study, we investigated whether social information can facilitate learning about a novel food source in *G. soricina*. Therefore, we conducted a classical demonstrator-observer dyad in which a naive focal bat had the task to feed from a novel food source in two different test situations, either together with a demonstrator bat that was already familiar with the food source, or, in order to control for social facilitation effects, together with another naive bat. We hypothesized that due to transmission of social information, focal bats would learn to exploit the novel food source faster when together with the knowledgeable demonstrator bat, as compared to focal bats in the control situation.

## Materials and methods

### Pre-experimental procedure

The study was conducted in the tropical dry forest of the Santa Rosa National Park, Guanacaste, Costa Rica (UTM: 16P 651137 1198498) in two periods from December 2014 to February 2015 and January to February 2016. We used 37 adult *G. soricina* (27 males, 10 females) that were caught from the wild with mist or hand nets and identified following the field key by Timm and LaVal (1998). We measured forearm length with a caliper (35.7 ± 0.9 mm, mean ± SD, n = 37) and body mass with a spring balance (10.3 ± 0.6 g, *n* = 37) (Pesola AG). Teeth characteristics for species identification were examined using a jewelry lens.

Prior to the experiment, focal bats were kept in a flight cage (Hexagon Screen House; Eureka) for at least one complete night (2.3 ± 1.2 nights) where they were fed with an artificial nectar solution (NektarPlus^®^ mixed with tap water 1:5, Nekton GmbH, Pforzheim, Germany) offered from a bowl that was placed slightly elevated on the floor. This nectar solution has a distinct odor, that is different from the odor of sulfur-based volatiles of many bat-pollinated flowers. Bats readily accepted this nectar solution and we did not have to release bats due to refusal to feed. With this pre-experimental captivity period, we allowed bats to habituate to being caged and thus avoided bats focusing on escape during the following experiment.

Demonstrator bats were kept inside the experimental cage for 2.3 ± 1.1 nights (Hexagon Screen House; Eureka) and trained to feed from the novel food source that was later used in the experiment. To minimize the number of used bats and reduce time for training periods in between experimental cycles, we trained a total of four demonstrator bats that were each used for up to three experimental cycles.

### Novel food source

**Fig. 1:**
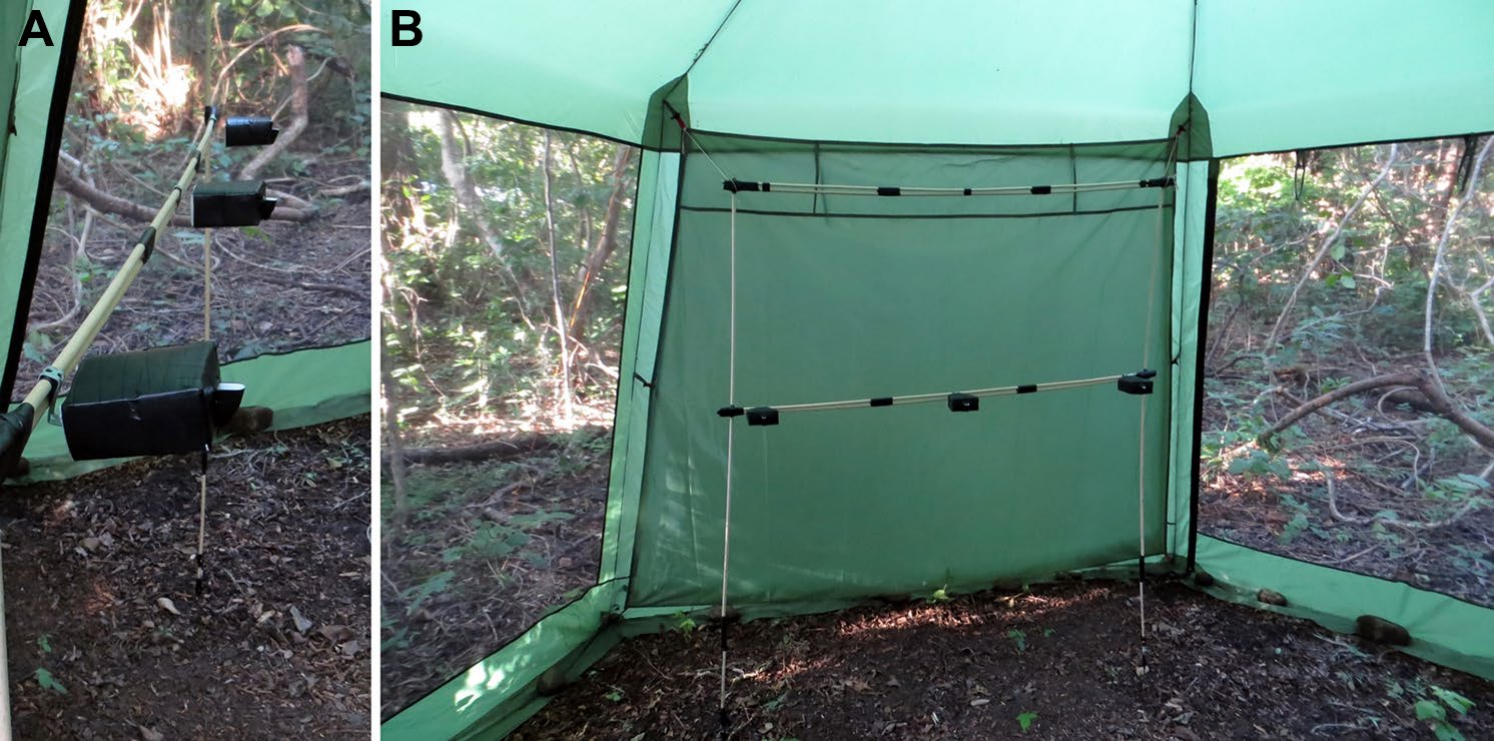
Black cuboidal boxes with small protruding opening (**A**) were presented as novel food source on an array inside the experimental cage (**B**)

As a novel food source, we used custom made black cuboidal boxes of 65 × 65 × 35 mm (l × w × h) that comprised a small protruding opening that allowed bats to access an internal sugar water reservoir with their tongues during hovering flight. The boxes differed substantially from bat-pollinated flowers (cf. Tschapka and Dressler 2002) and from potentially familiar artificial food sources like hummingbird feeders, as well as from the bowl of odorous NektarPlus® solution that was provided during the pre-experimental captivity period. Three of these boxes were mounted on an array at a height of 90 cm and at 50 cm distance from each other (Fig. 1A). The array was placed at one wall of the flight cage and thus very noticeably presented to the bats (Fig. 1B). During the experiment, one box was filled with sugar water (17% sucrose), while two boxes remained empty. With this design, we created a challenging foraging situation for focal bats, reflecting food sources that are not rewarding, like immature inflorescences or drained hummingbird feeders.

### Experimental cycle

**Fig. 2:**
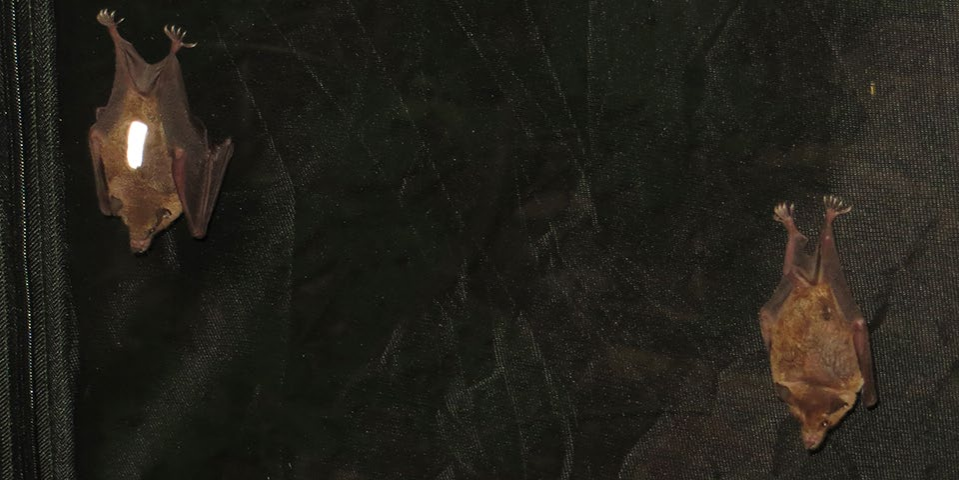
To allow a clear differentiation of bats from the video footage, focal bats (left) were marked with a reflective stripe on their back

Just before sunset (10.0 ± 9.9 min), bats were caught from their flight cages and placed in small extra cages without food (modified from PS FÅNGST, IKEA). One hour after sunset, we marked the focal bat by gluing a 2 cm reflective stripe on the tip of its back fur using superglue (Fig. 2) and released it to the experimental cage. Here it was allowed to get used to the marking and the experimental cage for 30 min. Subsequently, we mounted the boxes to the array and the experiment was started, either with releasing the demonstrator bat to the experimental cage in the social transmission situation, or with releasing another naïve bat in the control situation. The experiment lasted for 180 min, while bat flights towards the array were recorded by an infrared sensitive Camcorder (DCR-SR, Sony) under infrared illumination (2 HV L-IRC, Sony). By running the experiment at the beginning of the night, we ensured bats were motivated to search for food, but not food-deprived to the extent that they would resign foraging and enter torpor. We conducted 11 replications of each test situation during 22 evenings over two field seasons and used a total of 37 bats (social transmission situation: 11 focal bats, 4 demonstrator bats; control situation: 11 focal bats, 11 naïve bats). To ensure novelty of the food source, each focal and naïve bat was used in only one of the two test situations. Bat sexes were distributed as best as possible equally among the test situations.

### Analysis

From the video footage, bats’ behavior was scored until all bats had fed from the novel food source or the experimental time of 180 min had expired, summing up to a total of 48 h of scored video footage. We counted all examining approaches of bats towards boxes (i.e., approaching a box to closer than one body length combined with a change in direction or flight speed), all hovering flights while feeding, and all unsuccessful feeding attempts at empty boxes. After a focal bat had fed from the rewarding box, we scored an additional five minutes to check whether the newly learned food source was revisited.

To analyze the difference between the two test situations (i.e., social transmission situation with demonstrator present vs. control situation with another naïve bat present), we calculated the percentage of focal bats that successfully learned to feed from the novel food source within experimental time (success rate [%]), and the time span until bats first fed from the novel food source (learning time [min]). If a bat remained unsuccessful, we used the maximal experimental time of 180 min as a conservative approximation of their learning time (cf. Wright et al. 2020). Our design with one rewarding and two unrewarding food sources of the same shape allowed us to additionally gain information on whether focal bats rather learned via the location (i.e., food source visited by demonstrator), or by generalizing the demonstrated behavior towards the other, not demonstrated food sources. For this purpose, we analyzed the location of all first feeding attempts of focal bats.

**Fig. 3:**
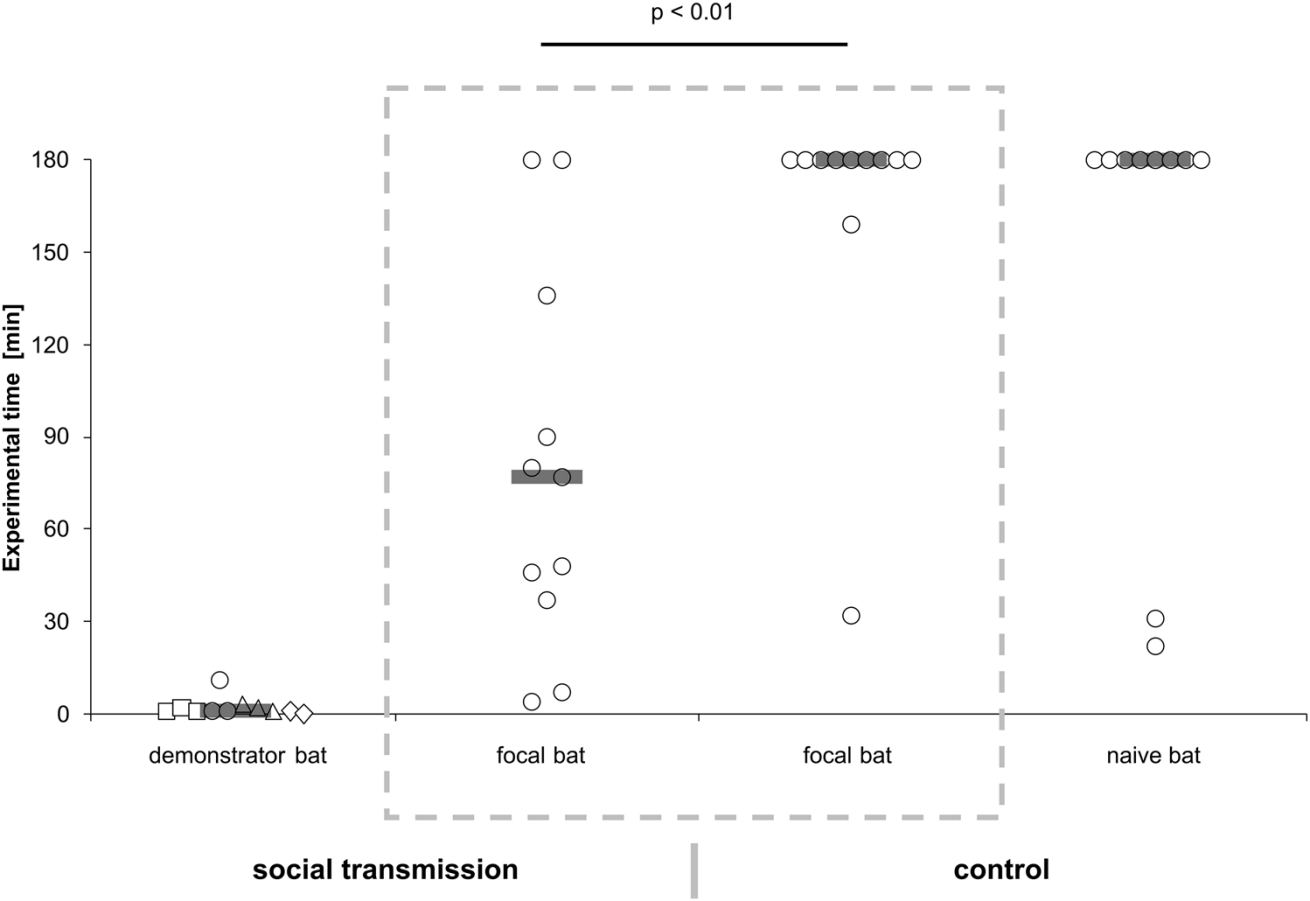
Learning time until bats first fed from the novel food source. In the social transmission situation, focal bats learned significantly faster than in the control situation (Wilcoxon rank sum test with continuity correction, *W* = 101, *p* < 0.01 (*α* = 0.05)). Solid lines depict medians and different symbols of demonstrator bats represent individuals

Statistical analysis was performed in R (v. 3.4.3, R Core Team 2017) using the Rcmdr package by Fox and Bouchet-Valat (2017). We compared learning time between both test situations using non-parametric Wilcoxon rank sum test with continuity correction, and success rate by Pearson’s Chi-squared test. Significance level (*α* = 0.05) was adjusted for multiple testing using sequential Bonferroni correction. The graph for Fig. 3 was created following Weissgerber et al. (2015).

## Results

In the social transmission situation, focal bats learned to feed from the novel food source after 81 ± 59 min (median: 77) with 9 of 11 focal bats being successful (success rate: 82%), while in the control situation, mean learning time was at 165 ± 42 min (median: 180), twice as long with only 2 of 11 focal bats successfully feeding within the experimental time (success rate: 18%) (learning time: Wilcoxon rank sum test with continuity correction, *W* = 101, *p* < 0.01 (*α* = 0.05); success rate: Pearson’s Chi-squared test, *χ*^2^ = 8.9091, *df* = 1, *p* < 0.01 (*α* = 0.025)) (Fig. 3). Demonstrator bats visited the food source for the first time 2 ± 3 min after starting the experiment (median: 1) and performed a total of 27.5 ± 18.6 feeding visits (median: 20) before focal bats first fed from the food source or experimental time expired. Each feeding visit lasted for about one second. In both test situations, focal bats remained active throughout experimental time and regularly performed flights inside the cage (Table 1). In the social transmission situation, 60% of first feeding attempts by focal bats were performed at the demonstrated rewarding food source (chance probability: 33%; control situation: 40%). All successful focal bats revisited the novel food source for feeding and, after feeding, five also attempted to feed from a nearby empty box. In the control situation, naive bats behaved similarly to focal bats (success rate: 18%, learning time: 152 ± 59 min (median: 180)).

**Table 1:** Examining approaches of focal bats towards boxes, feeding attempts at unrewarding boxes and the location of first feeding attempts before successfully feeding or expiration of experimental time

	Social transmission	Control
Examining approaches	5.1 ± 4.3 (median: 4)	6.5 ± 2.9 (median: 6)
Unrewarding feeding attempts	1.1 ± 1.5 (median: 0)	0.6 ± 1.2 (median: 0)
First feeding attempt unrewarding	4	3
First feeding attempt rewarding	6	2
Successfully feeding	9	2

## Discussion

In the presence of a knowledgeable demonstrator, focal bats learned to exploit the novel food source faster and more often than compared to the control situation, which implicates a transmission of information between bats. Since we never tested focal bats alone in our setup, we cannot assess whether the mere presence of a conspecific may have additionally facilitated the learning process, as social facilitation was reported to be beneficial for foraging frugivorous bats (Wright et al. 2020), and also flower-visiting bats are probably bolder and more readily interacting with novel objects if conspecifics are present (Hörmann et al. 2021). Although our experiment was not designed to reveal the actual mechanism behind the observed social transmission, it is likely that the demonstrators’ behavior of approaching and feeding guided the focal bats’ attention towards the array and the novel food source, or that focal bats used the demonstrators’ hovering flights as a cue for the presence of food in this location (cf. local enhancement, Hoppitt and Laland 2013). Our additionally gained data on the location of first feeding attempts may point in this direction. Although the number of first feeding attempts in the control situation is too small for a conclusive comparison, the high probability of focal bats in the social transmission situation to perform their first feeding attempts on the demonstrated rewarding food source may indicate that bats learned rather via the demonstrated location, than by directly generalizing the demonstrator’s behavior to the unrewarding food sources of same shape. Such location-dependent social learning strategies, for instance via following behavior (e.g., Wilkinson 1992) or by eavesdropping on conspecific feeding buzzes (e.g., Barclay 1982) are readily applied by bats searching for new locations of already known food sources. However, flower-visiting bats should also be well suited to apply location-dependent strategies when socially learning about actual food characteristics, since nectar sources such as flowers are usually not removed by a demonstrators’ visit. After the initial learning about a particular novel food source at one location, there is no doubt that flower-visiting bats are able to generalize this knowledge and recognize the same type of food source also at different locations (von Helversen 2004; Thiele and Winter 2005). This is in line with our observation that within 5 min after successfully feeding, five focal bats also attempted to feed on one of the nearby unrewarding boxes. In contrast, for predatory or frugivorous species learning about novel food sources via location is probably not as suitable, since prey or fruits are usually removed by the act of feeding, obliging these bats to socially learn via actual food cues such as sound (e.g., Page and Ryan 2006) or odor (e.g., Ratcliffe and ter Hofstede 2005; O’Mara et al. 2014).

In anthropogenically altered environments, flower-visiting bats can be observed to expand their ecological niche by incorporating novel food sources into their dietary repertoire. For example, *G. soricina* thrive in agricultural monocultures by exploiting flowers of old-world banana plants (Murphy et al. 2016; Alpízar et al. 2020), the usage of introduced ornamental plants may enable bats to occur in urban areas (Kruszynski et al. 2016; da Silva et al. 2017; Pellón et al. 2021) and groups of flower-visiting lesser long-nosed bats (*Leptonycteris yerbabuenae*) are only able to migrate through certain desert locations because they learned to utilize artificial hummingbird feeders at urban homes (Buechner and Sidner 2013). Such an adoption of novel food sources that are not part of the bats’ natural dietary repertoire can also be observed in species with different feeding habits. Insectivorous long-fingered bats (*Myotis capaccinii*) may have shifted in northern Israel from insectivory to semi-piscivory after a small fish species was introduced for mosquito control (Levin et al. 2006). Common vampire bats (*Desmodus rotundus*) parasitize livestock in rural areas (Bobrowiec et al. 2015) and on pets in urban areas (Rosa et al. 2013), while frugivorous bats may take advantage of cultivated crops (Parry-Jones and Augee 1991). However, there is no information on which degree the utilization of these food sources is driven by social learning, thus representing a socially transmitted behavior or in some regions even a local tradition, or whether it rather represents an opportunistic feeding behavior independently invented by multiple individuals (van Schaik 2010).

Progressively expanding their natural dietary repertoire with novel food sources is central for young bats during the transition from parental care to independent life, and a decisive role of social learning in this period is frequently suggested (Wright 2016; Rose et al. 2020). However, experiments on vertical social learning of foraging behavior in bats are scarce. Although the presence of experienced adults facilitated learning about foraging in young frugivorous and insectivorous bats (Wright et al. 2011; Ganesh et al. 2016), other studies on insectivorous and nectarivorous bats failed to provide evidence for a vertical transmission of foraging-related information from parents to offspring (Ripperger et al. 2019; Rose et al. 2019, 2020). For adult bats, several experiments in captivity demonstrated the application of social learning strategies when learning about novel food sources or dietary preferences (e.g., Ratcliffe and ter Hofstede 2005; Page and Ryan 2006; Wright et al. 2011; Jones et al. 2013; Ramakers et al. 2016), but comparable field experiments with free ranging bats are scarce (O’Mara et al. 2014) or rather focusing on learning about the spatial distribution of food (e.g., Barclay 1982; Wilkinson 1992; Ripperger et al. 2019; Rose et al. 2020). However, conclusions from mere lab or flight cage experiments have to be drawn carefully, since experiments in captivity often provide conditions that may rarely appear in nature, including forced spatial proximity of knowledgeable and learning individuals or a lack of alternative food sources. Therefore, an observed social transmission in artificial demonstrator-observer dyads may not be sufficient evidence for the respective transmission chains occurring also under natural conditions (Laland and Plotkin 1990). Further, all socially transmitted behaviors can be learned individually, as also focal bats in our control situation managed to feed from the novel food source, and every social transmission chain observed in animals was inevitably initiated at one point (Reader and Laland 2003). In this regard, it would be important to investigate the cues that facilitate an initial individual discovery of novel food sources in the wild. For instance, in the case of flower-visiting bats and hummingbird feeders, it seems conceivable that an initial discovery could be facilitated by the scent of fermenting sugar water, a scent bats may already be familiar with from natural nectar sources.

In conclusion, our study adds flower-visiting bats to the list of bats that are capable of using social information to learn about novel food sources, but relevance for and incidence in free-living bats remains unclear. Further studies should move from captivity to experiments with free ranging bats and investigate social learning about foraging behavior under natural conditions. Hereby, anthropogenically introduced food sources that are not part of the bats’ natural dietary repertoire may represent valuable study objects.

## Supplementary Information

Below is the link to the electronic supplementary material.

Supplementary file1 (PDF 72 KB)

Supplementary file2 (AVI 4796 KB)

Supplementary file3 (AVI 7047 KB)

Supplementary file4 (AVI 5117 KB)

Supplementary file5 (AVI 2556 KB)

Supplementary file6 (AVI 7694 KB)

## Data Availability

The dataset collected and analyzed during the current study is available from the corresponding author on reasonable request.
