# Supplementary material for: Social information facilitates learning about novel food sources in adult flower-visiting bats
Source: Anim Cogn. 2023 Jul 8;26(5):1635–42. doi: 10.1007/s10071-023-01807-9 (PMC10442281; doi:10.1007/s10071-023-01807-9)
Supplement: Supplementary file 1 — Supplementary file1 (PDF 72 KB) [file 10071_2023_1807_MOESM1_ESM.pdf]

Video1.avi
social transmission situation

Focal bat (marked by a reflective stripe) approaching the feeding demonstrator bat twice at the rewarding novel food source (left) before inspecting and feeding from it. Subsequently, it also attempts to feed from the unrewarding novel food sources of same shape (middle and right).

Video2.avi
social transmission situation

Focal bat approaching the feeding demonstrator bat and feeding from the rewarding novel food source (middle).

Video3.avi
social transmission situation

Focal bat approaching the feeding demonstrator bat and performing a feeding attempt at an unrewarding novel food source (middle) before feeding from the rewarding novel food source (left).

Video4.avi
control situation

Focal bat inspecting the array without recognizing potential food sources.

Video5.avi
control situation

Focal bat inspecting the array and learning to feed from the rewarding novel food source (right) without demonstrator.
